# Altered brain functional networks in schizophrenia with persistent negative symptoms: an activation likelihood estimation meta-analysis

**DOI:** 10.3389/fnhum.2023.1204632

**Published:** 2023-10-26

**Authors:** Tingting Zhu, Zengxiu Wang, Weifeng Wu, Yuru Ling, Zixu Wang, Chao Zhou, Xinyu Fang, Chengbing Huang, Chunming Xie, Jiu Chen, Xiangrong Zhang

**Affiliations:** ^1^Department of Psychiatry, The Third People’s Hospital of Huai’an, Huaian, Jiangsu, China; ^2^Department of Geriatric Psychiatry, The Affiliated Brain Hospital of Nanjing Medical University, Nanjing, China; ^3^Department of Hepatology, The Second Hospital of Nanjing, Nanjing University of Chinese Medicine, Nanjing, China; ^4^Department of Neurology, Affiliated Zhongda Hospital, School of Medicine Southeast University, Nanjing, China; ^5^Institute of Neuropsychiatry, The Affiliated Brain Hospital of Nanjing Medical University, Nanjing, China; ^6^The Affiliated Xuzhou Oriental Hospital of Xuzhou Medical University, Xuzhou, Jiangsu, China

**Keywords:** persistent negative symptoms, functional connectivity, gray matter volume, default mode network, central executive network, salience network

## Abstract

**Objective:**

To investigate brain structural and functional characteristics of three brain functional networks including default mode network (DMN), central executive network (CEN), and salience network (SN) in persistent negative symptoms (PNS) patients.

**Methods:**

We performed an activation likelihood estimation (ALE) meta-analysis of functional connectivity (FC) studies and voxel-based morphometry (VBM) studies to detect specific structural and functional alterations of brain networks between PNS patients and healthy controls.

**Results:**

Seventeen VBM studies and twenty FC studies were included. In the DMN, PNS patients showed decreased gray matter in the bilateral medial frontal gyrus and left anterior cingulate gyrus and a significant reduction of FC in the right precuneus. Also, PNS patients had a decrease of gray matter in the left inferior parietal lobules and medial frontal gyrus, and a significant reduction of FC in the bilateral superior frontal gyrus in the CEN. In comparison with healthy controls, PNS patients exhibited reduced gray matter in the bilateral insula, anterior cingulate gyrus, left precentral gyrus and right claustrum and lower FC in these brain areas in the SN, including the left insula, claustrum, inferior frontal gyrus and extra-nuclear.

**Conclusion:**

This meta-analysis reveals brain structural and functional imaging alterations in the three networks and the interaction among these networks in PNS patients, which provides neuroscientific evidence for more personalized treatment.

Systematic Review RegistrationThe PROSPERO (https://www.crd.york.ac.uk/PROSPERO/, registration number: CRD42022335962).

## 1. Introduction

Negative symptoms are related to severe impairment in social function and have a negative impact on the treatment and prognosis of schizophrenia (Galderisi et al., 2018). Negative symptoms include blunted affect, anhedonia, alogia, avolition, and a sociality (Kirkpatrick et al., 2006; Buchanan, 2007). Negative symptoms recently have been mainly divided into the following two subtypes (Szendi et al., 2017). One is deficit syndrome, which refers to primary or idiopathic persistent negative symptoms. Another is the concept of Persistent negative symptoms (PNS), described as primary or secondary negative symptoms of moderate or worse severity lasting more than 6 months after the first episode of psychosis (Carpenter et al., 1988; Castellon et al., 1994), and demonstrating defined threshold levels of positive symptoms, depression, and extrapyramidal side effects during the stable phase of schizophrenia (Buchanan, 2007). The PNS is considered as a broader concept than deficit syndrome and is not specific for diagnosis and therefore more suitable for clinical trials. Previous studies have shown that the duration of untreated psychosis is associated with the development of PNS and can be considered a risk factor (Galderisi et al., 2013; González-Valderrama et al., 2017) and it clinically represents an unmet therapeutic need in many cases (Kirkpatrick et al., 2006). Therefore, effective diagnosis and appropriate intervention for PNS patients are essential.

Previous studies have revealed anomalous connectivity in several functional networks, including the default mode network (DMN) (Yang et al., 2019; Fan et al., 2020), central executive network (CEN) (Chen et al., 2016), and salience network (SN) (Huang et al., 2020), which have been considered as potential neural network foundation for psychopathology and abnormal cognition and emotion (Menon, 2011). The DMN includes the medial prefrontal cortex, posterior cingulate cortex, precuneus, medial and inferior temporal lobes, and bilateral inferior parietal lobules (IPL) (Buckner et al., 2008, 2011; Yeo et al., 2011). This network is usually active when participants are not working on any specific task and inactive during effortful cognitive tasks (Raichle et al., 2001; Greicius et al., 2009). It is also manages the monitoring associated with internal generative processes, including autobiographical memory recollection, self-monitoring, and internal and external cognition (Buckner et al., 2008). The CEN includes the superior frontal gyrus (SFG), dorsolateral prefrontal cortex and posterior parietal cortices, and is involved in goal-directed/externally oriented tasks and the working memory process. The SN, anchored on the insula and anterior cingulate gyrus (ACG), plays an important role in cognitive control by focusing on motivational salience stimuli and employing suitable functional brain-behavior networks to regulate actions (Menon, 2011; Peters et al., 2016). Exploring structural and functional changes within these three brain networks in patients with PNS will help provide a neuroimaging basis for designing targeted and effective interventions.

Recent advances in neuroimaging technology have accumulated substantial evidence regarding functional and structural alterations in these brain networks in schizophrenia. Voxel-based morphometry (VBM) has been broadly accepted to detect structural alterations in brain networks, and it can provide an unbiased method for estimating regional gray matter volume (GMV) (Ashburner and Friston, 2000). Independent component analysis and seed-based resting state functional connectivity (rs-FC) can reflect connectivity between different brain regions or networks (Cole et al., 2010). These two imaging techniques are better comparable in independent study settings and populations, and are not affected by task paradigms (Shehzad et al., 2009; Liu et al., 2020), so this study conducted a meta-analysis of structural (volumetric) and resting-state functional magnetic resonance imaging (rs-MRI) findings. Results of previous VBM studies showed reduced GMV within the DMN, CEN, and SN in schizophrenia (Honea et al., 2008; Fornito et al., 2009; Schuster et al., 2012; Kim et al., 2017). Meanwhile, the accumulated evidence has suggested that brain activity of the three networks is disrupted in schizophrenia, but the results are inconsistent (Chen et al., 2016; Hare et al., 2019; Yang et al., 2019; Fan et al., 2020; Huang et al., 2020). Few studies have been conducted on PNS patients and the findings are inconsistently arising from the use of different acquisition, processing, analysis techniques and different sample characteristics (Benoit et al., 2012; İnce and Üçok, 2018). It is obvious that more studies with larger sample sizes are needed in the future to investigate brain structural and functional alterations in PNS patients.

The main findings of VBM and FC studies were summarized in some systematic reviews and meta-analyses, most of which investigated a widespread tendency of reduced GMV and large-scale brain network connectivity in schizophrenia (Ellison-Wright et al., 2008; Pettersson-Yeo et al., 2011; Haijma et al., 2013; Kambeitz et al., 2016; Dong et al., 2018). Furthermore, several studies (Brady et al., 2019; Gao et al., 2020), and meta-analyses (Li et al., 2018; Chee et al., 2020) investigating the neuroanatomy and neurological relevance of psychiatric symptoms have shown that demonstrating that GMV and FC aberrations in schizophrenia are associated with negative symptoms severity. Several articles have revealed structural and functional alterations in these core networks in PNS patients (Benoit et al., 2012; Bodnar et al., 2014; İnce and Üçok, 2018). However, only one meta-analysis has found reduced GMV in the brain regions of the reward network, especially the left caudate nucleus in patients with PNS compared with HC (Li et al., 2018). Hence, it is necessary to conduct a meta-analysis of whole-brain VBM and FC studies to assess certain structural and functional changes in DMN, CEN and SN in PNS patients.

## 2. Methods

### 2.1. Literature search

**Figure 1:**
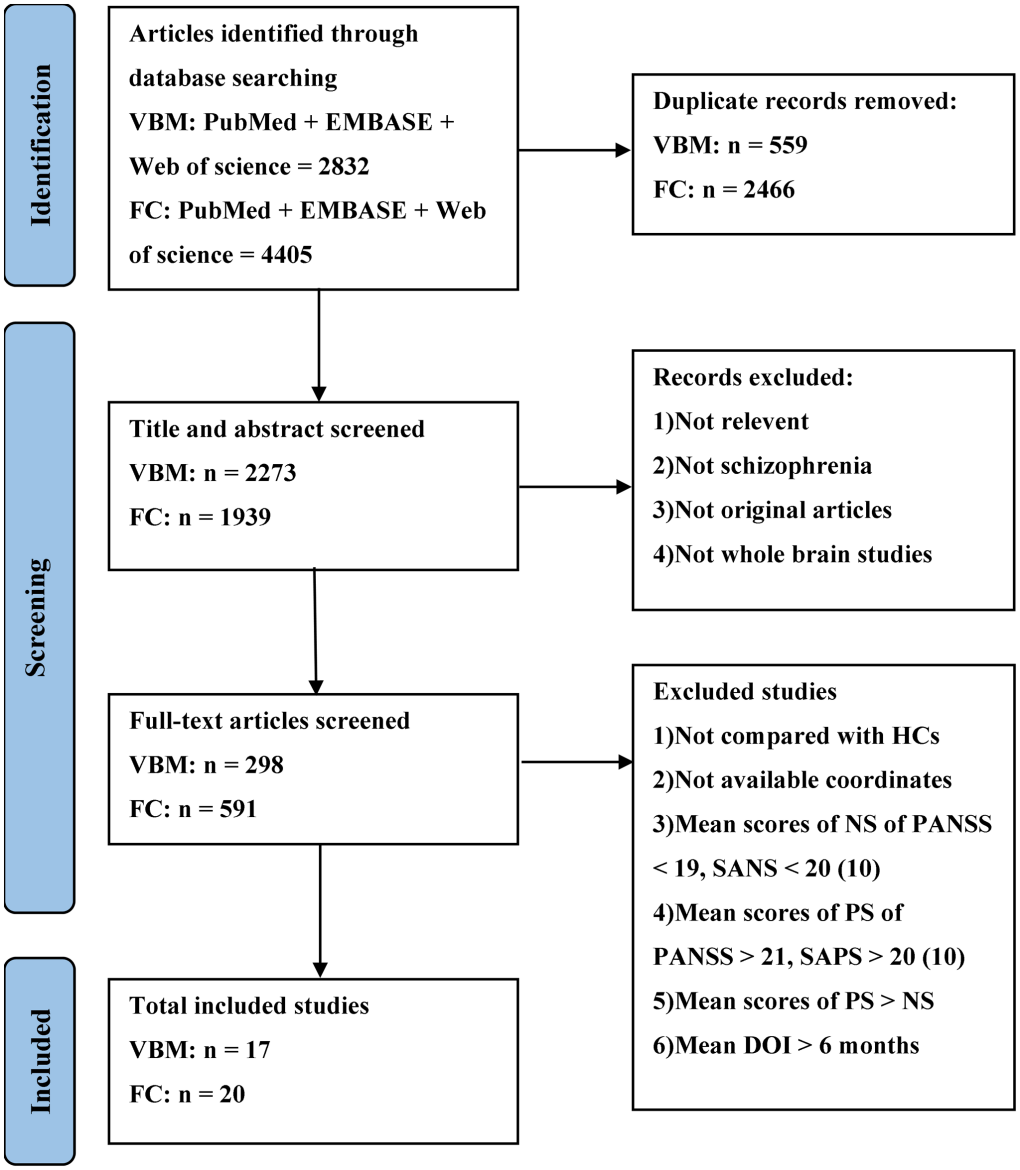
The selection procedure for the inclusion of empirical studies.

The meta-analysis was preregistered on the PROSPERO (https://www.crd.york.ac.uk/PROSPERO/, registration number: CRD42022335962) and performed in accordance with the PRISMA statement (Moher et al., 2009). Structural and functional imaging studies related to DMN, SN and CEN were retrieved from the PubMed, Web of Science and EMBASE databases up to July 2023. The search keywords were “schizophrenia” and “default mode network/central executive network/salience network” and “gray matter” and “voxel-based morphometry”; “schizophrenia” and “default mode network/central executive network/salience network” and “functional connectivity” and “Functional Magnetic Resonance Imaging/functional MRI/fMRI.” Figure 1 shows the selection procedure for the inclusion of empirical studies.

### 2.2. Eligible criteria

The studies included in this meta-analysis were required to meet the following requirements: (1) Negative symptoms of patients must be at least mild or moderate in severity, and assessed by a proven negative symptom scale such as the Positive and Negative Syndrome Scale (PANSS), Scale for the Assessment of Negative Symptoms (SANS), Negative Symptoms Assessment, and Brief Negative Symptom Scale (Kay et al., 1987; Andreasen, 1989; Kirkpatrick et al., 2011). (2) The duration of illness is at least 6 months. (3) The included studies need to use the whole-brain analysis method (VBM or FC) to investigate the brain imaging differences between schizophrenia and healthy subjects and use the Montreal Neurologic Institute (MNI) or Talairach system to report the results. (4) All studies compared PNS patients with healthy controls (HCs), and we adopted the following exclusion criteria formulated by Li et al. (2018) to identify the relevant studies: (1) The patient’s mean PANSS negative score was less than 19, and the mean PANSS positive subscale score was greater than 21. (2) The patient’s mean SANS total score was less than 20, and the mean Scale for the Assessment of Positive Symptoms total score was greater than 20. (3) The patient’s mean positive symptom scores was greater than negative symptom scores.

### 2.3. Data extraction and quality assessment

All data were extracted separately by two authors, with a third author resolving any inconsistencies before the final analysis. First, we chose DMN, CEN, and SN related articles. Second, all aberrant brain areas retrieved from the articles were grouped as one of three brain networks (DMN, CEN, or SN). Third, we extracted the coordinates that we needed from whole-brain VBM/FC studies. Finally, we extracted the general characteristics of each study, including the first author, year of publication, sample size, sex of subjects, illness duration, and assessment scale were extracted as the basic data. We used a 12-point checklist to assess the methodological quality of the included studies (Brambilla et al., 2003), and case studies with scores above 6.0 were included in our meta-analysis.

### 2.4. Activation likelihood estimation

We used Ginger activation likelihood estimation (ALE) version 2.3.6^1^ to run the ALE algorithm and perform a voxel-based meta-analysis of neuroimaging data (Eickhoff et al., 2017). Structural and functional studies were analyzed separately, and we extracted coordinates of increased FC in schizophrenia compared with healthy subjects and decreased FC in schizophrenia compared with healthy subjects in the included study for meta-analysis, and divided the former into FC increasing group and the latter into FC decreasing group. First, we turned the activation coordinates reported in the study in Talairach space into MNI space. Then, we collected the coordinates of these three specific networks containing brain regions and followed the software guide to obtain the final cluster. The present study performed family-wise error (FWE) correction at *p* < 0.05 for cluster-level inference, and threshold at voxel level *p* < 0.01 for cluster-forming, with 1,000 permutations. Lastly, we present the ALE maps through the DPABI software (Yan et al., 2016).^2^

## 3. Results

### 3.1. Search results

According to the search formula, 2,832 VBM articles and 4,405 FC articles were retrieved from the three databases, of which 559 and 2,466 duplicate articles were retrieved, respectively. After rigorous screening, a total of 17 VBM studies and 20 FC studies were selected. More detailed information on each of the included studies is shown in Tables 1, 2. For the VBM-meta-analysis, we identified 15 experiments with the DMN (Paillère-Martinot et al., 2001; Sigmundsson et al., 2001; Salgado-Pineda et al., 2004; Jayakumar et al., 2005; Bassitt et al., 2007; Koutsouleris et al., 2008; Meisenzahl et al., 2008; Herold et al., 2009; Whitford et al., 2009; Huang et al., 2015; Poletti et al., 2016; Kuroki et al., 2017; Szendi et al., 2017; Spalthoff et al., 2018; Neugebauer et al., 2019), 5 experiments with the CEN (Jayakumar et al., 2005; Bassitt et al., 2007; Koutsouleris et al., 2008; Meisenzahl et al., 2008; Herold et al., 2009), and 15 experiments with the SN (Paillère-Martinot et al., 2001; Sigmundsson et al., 2001; Salgado-Pineda et al., 2004; Jayakumar et al., 2005; Bassitt et al., 2007; Koutsouleris et al., 2008; Meisenzahl et al., 2008; Herold et al., 2009; Anderson et al., 2015; Poletti et al., 2016; Kim et al., 2017; Kuroki et al., 2017; Szendi et al., 2017; Spalthoff et al., 2018; Neugebauer et al., 2019). For the FC-meta-analysis, we identified 17 experiments with the DMN (Bluhm et al., 2007; Wolf et al., 2011; Fan et al., 2013; Chang et al., 2014; Manoliu et al., 2014; Zhuo et al., 2014, 2017; Alonso-Solís et al., 2015; Xu et al., 2015; Zhou et al., 2015; Chen et al., 2016; Penner et al., 2016, 2018a,b; Peters et al., 2017; Sharma et al., 2018; Dong et al., 2019), 7 experiments with the CEN (Chang et al., 2014; Manoliu et al., 2014; Zhuo et al., 2014; Penner et al., 2016, 2018a,b; Peters et al., 2017), and 10 experiments with the SN (Wolf et al., 2011; Manoliu et al., 2014; Wang et al., 2015; Xu et al., 2015; Zhou et al., 2015; Chen et al., 2016; Penner et al., 2016, 2018a,b; Peters et al., 2017) reporting decreased FC in the PNS group relative to the HC group. In addition, 6 experiments with the DMN (Wolf et al., 2011; Chang et al., 2014; Alonso-Solís et al., 2015; Wang et al., 2015, 2016; Liu et al., 2018), 3 experiments with the CEN (Wolf et al., 2011; Manoliu et al., 2014; Penner et al., 2018a), and 2 experiments with the SN (Manoliu et al., 2014; Chen et al., 2016) reported that FC was increased in the PNS patients. The quality assessment of the included studies ranged from low scores of 10.5 and moderate scores of 11–11.5 to high scores of 12. The result of the quality assessment and the areas involved in core brain networks in each study are detailed in Supplementary material.

**Table 1:** Description of the VBM studies included in the meta-analysis.

Study	SZ			HC			NS scale	NS	PS	DOI (years)
	No.	M/F	Age (mean)	No.	M/F	Age (mean)				
Neugebauer et al. (2019)	18	11/7	36.94	19	12/7	35.79	PANSS	23.61	15.33	12.58
Spalthoff et al. (2018)	51	34/17	35.18	102	69/33	33.15	SANS	42.45	19.38	8.8
Szendi et al. (2017)	8	6/2	34	13	6/7	34	PANSS	27.5	17.5	13
Kuroki et al. (2017)	15	15/0	44.1	23	23/0	36.8	PANSS	19.7	14.3	18
Kim et al. (2017)	22	12/10	31.7	22	12/10	31.6	PANSS	21.1	18.4	9.2
Poletti et al. (2016)	96	67/29	37.24	136	68/68	33.31	PANSS	20.41	17.62	12.61
Huang et al. (2015)	18	9/9	22.67	18	9/9	25.06	PANSS	22.06	18.61	1.04
Anderson et al. (2015)	15	13/2	34.3	20	17/3	33.3	PANSS	20	13	11.4
Whitford et al. (2009)	31	20/11	19.3	21	12/9	19.6	PANSS	20	18	0.53
Herold et al. (2009)	18	11/7	28.7	21	11/10	27.4	PANSS	19.6	14.2	3.4
Meisenzahl et al. (2008)	93	67/26	28.2	177	123/54	31.5	PANSS	20.4	19.9	0.76
Koutsouleris et al. (2008)	175	130/45	31.7	177	123/54	31.5	PANSS	22.3	19	1.6
Bassitt et al. (2007)	50	38/12	31.7	30	21/9	31.2	PANSS	19.8	12.9	11.4
Jayakumar et al. (2005)	18	9/9	24.9	18	9/9	25.7	PANSS	23	19	0.86
Salgado-Pineda et al. (2004)	14	7/7	25.05	14	7/7	25.14	SANS	21.21	9.78	1
Sigmundsson et al. (2001)	27	26/1	34.9	27	25/2	32.2	PANSS	25	14.7	13.9
Paillère-Martinot et al. (2001)	20	20/0	29	20	20/0	26	PANSS	27.6	17.3	10

SZ, schizophrenia; HC, healthy control; VBM, voxel-based morphometry; DOI, duration of illness; M/F, male/female; PANSS, Positive and Negative Syndrome Scale; SANS, Scale for the Assessment of Negative Symptom; NS, negative symptoms; PS, positive symptoms.

**Table 2:** Description of the FC studies included in the meta-analysis.

Study	SZ			HC			NS scale	NS	PS	DOI
	No.	M/F	Age (mean)	No.	M/F	Age (mean)				
Dong et al. (2019)	96	66/30	39.8	122	81/41	38.0	PANSS	20.73	13.44	15.1
Sharma et al. (2018)	34	22/12	29.32	19	12/7	31.53	SANS	31.74	15	3.73
Penner et al. (2018a)	24	21/3	23.2	24	12/12	23.8	SANS	22.5	10.3	13.7
Penner et al. (2018b)	24	21/3	23.2	24	12/12	23.8	SANS	22.5	10.3	13.7
Liu et al. (2018)	21	15/6	30.95	21	14/7	31.43	PANSS	22.19	11.62	4.74
Zhuo et al. (2017)	95	54/41	33.6	93	45/48	33	PANSS	20.3	17.1	10.12
Peters et al. (2017)	21	10/11	34.05	21	10/11	33.49	PANSS	21.14	19.4	7.15
Wang et al. (2016)	31	19/12	20.61	37	18/19	20.76	PANSS	20.32	20	0.43
Penner et al. (2016)	24	21/3	23.2	24	12/12	23.8	SANS	22.5	10.3	1.14
Chen et al. (2016)	46	32/14	41.54	46	24/22	39.05	PANSS	20.61	12.52	16.27
Zhou et al. (2015)	91	51/40	33.8	100	45/55	33.3	PANSS	20	16.6	10
Xu et al. (2015)	66	38/28	33	76	38/38	33	PANSS	21.1	17	9.5
Wang et al. (2015)	94	52/42	33.6	102	45/57	33.4	PANSS	20.3	16.6	10
Alonso-Solís et al. (2015)	19	13/6	40.05	20	13/7	37.75	PANSS	21.47	17.89	16.11
Zhuo et al. (2014)	95	54/41	33.6	93	45/48	33	PANSS	20.3	17.1	10.12
Chang et al. (2014)	25	13/12	25.36	25	14/11	25.48	PANSS	21.39	18.73	1.53
Manoliu et al. (2014)	18	9/9	35.33	20	9/11	34	PANSS	19.94	18.06	7
Fan et al. (2013)	27	16/11	39.7	15	8/7	41.4	PANSS	20.9	18.9	16.5
Wolf et al. (2011)	10	6/4	36.5	14	7/7	33.7	PANSS	22	16	9.9
Bluhm et al. (2007)	17	14/3	33.54	17	14/3	30.94	SANS	20.35	9.06	9.78

SZ, schizophrenia; HC, healthy control; FC, functional connectivity; DOI, duration of illness; M/F, male/female; PANSS, Positive and Negative Syndrome Scale; SANS, Scale for the Assessment of Negative Symptom; NS, negative symptoms; PS, positive symptoms.

### 3.2. Meta-analysis results

**Figure 2:**
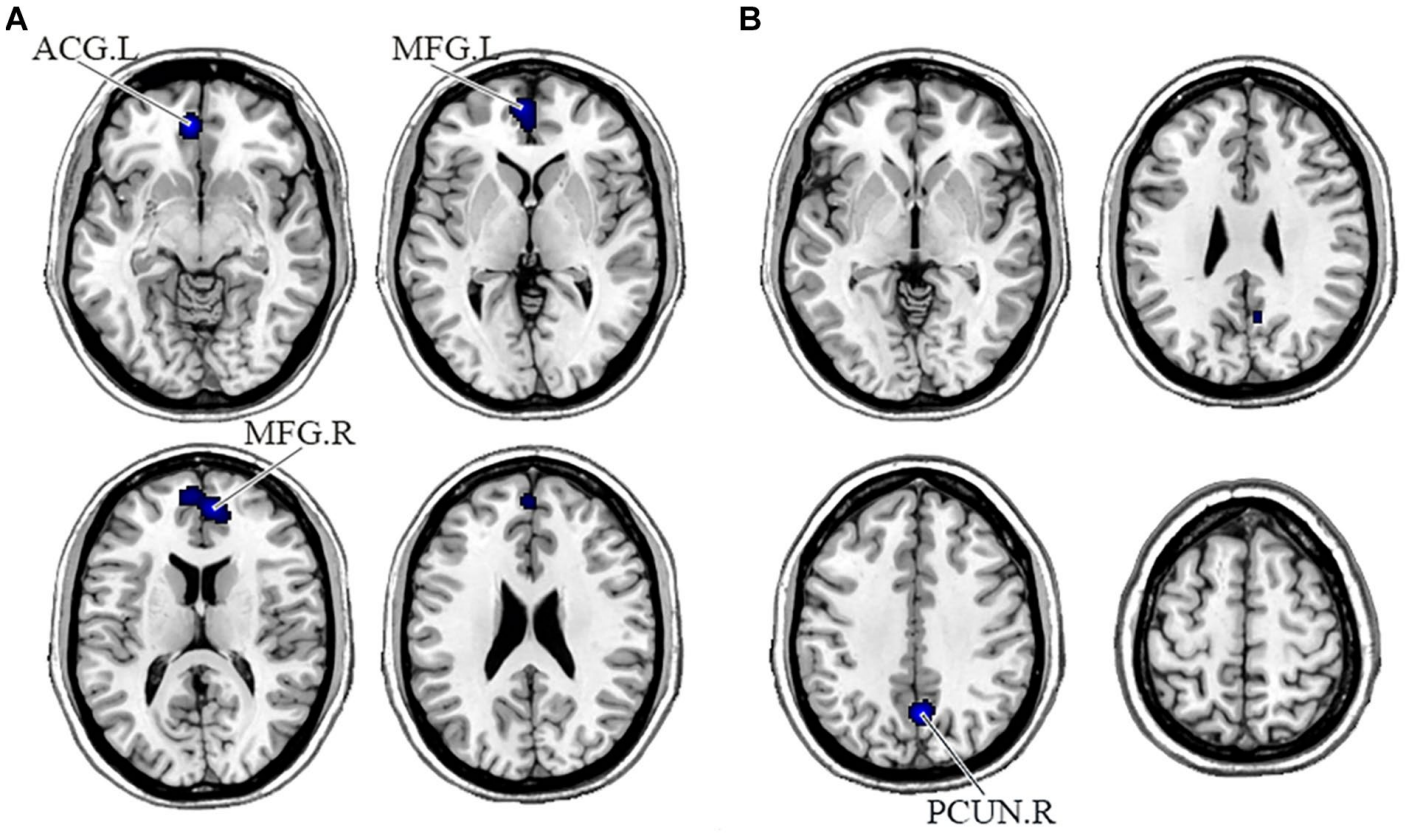
Displayed are significant results from the meta-analysis of the DMN studies. **(A)** Gray matter reduction in the DMN in PNS patients relative to HCs. **(B)** Areas showing lower FC in the DMN in PNS patients relative to HCs. FC, functional connectivity; PNS, Persistent negative symptoms; HCs, healthy controls; DMN, default mode network; ACG, anterior cingulate gyrus; MFG, medial frontal gyrus; PCUN, precuneus; R, right; L, left.

**Figure 3:**
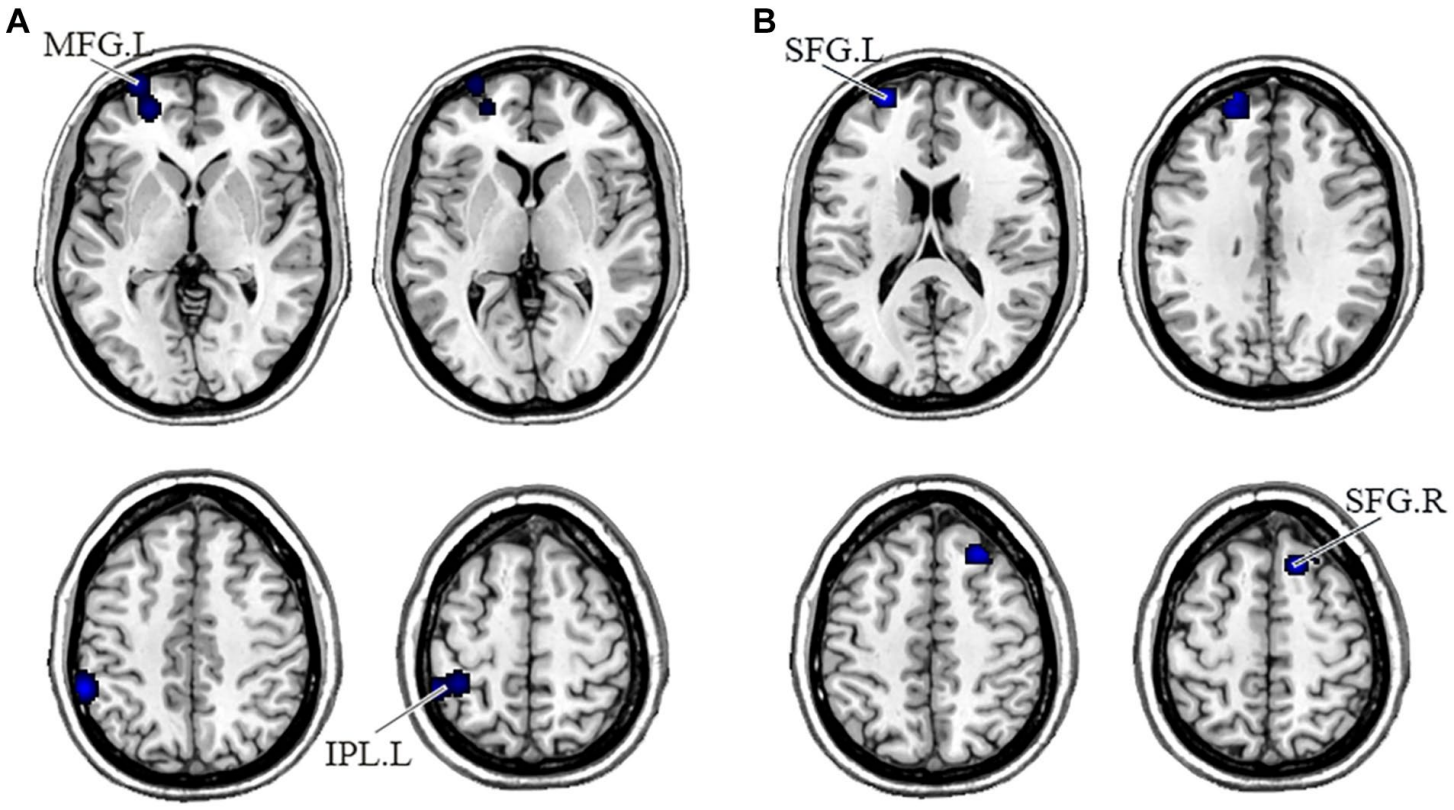
Displayed are significant results from the meta-analysis of the CEN studies. **(A)** Gray matter reduction in the CEN in PNS patients relative to HCs. **(B)** Areas showing lower FC in the CEN in PNS patients relative to HCs. FC, functional connectivity; PNS, Persistent negative symptoms; HCs, healthy controls; CEN, central executive network; MFG, medial frontal gyrus; IPL, inferior parietal lobule; SFG, superior frontal gyrus; R, right; L, left.

**Figure 4:**
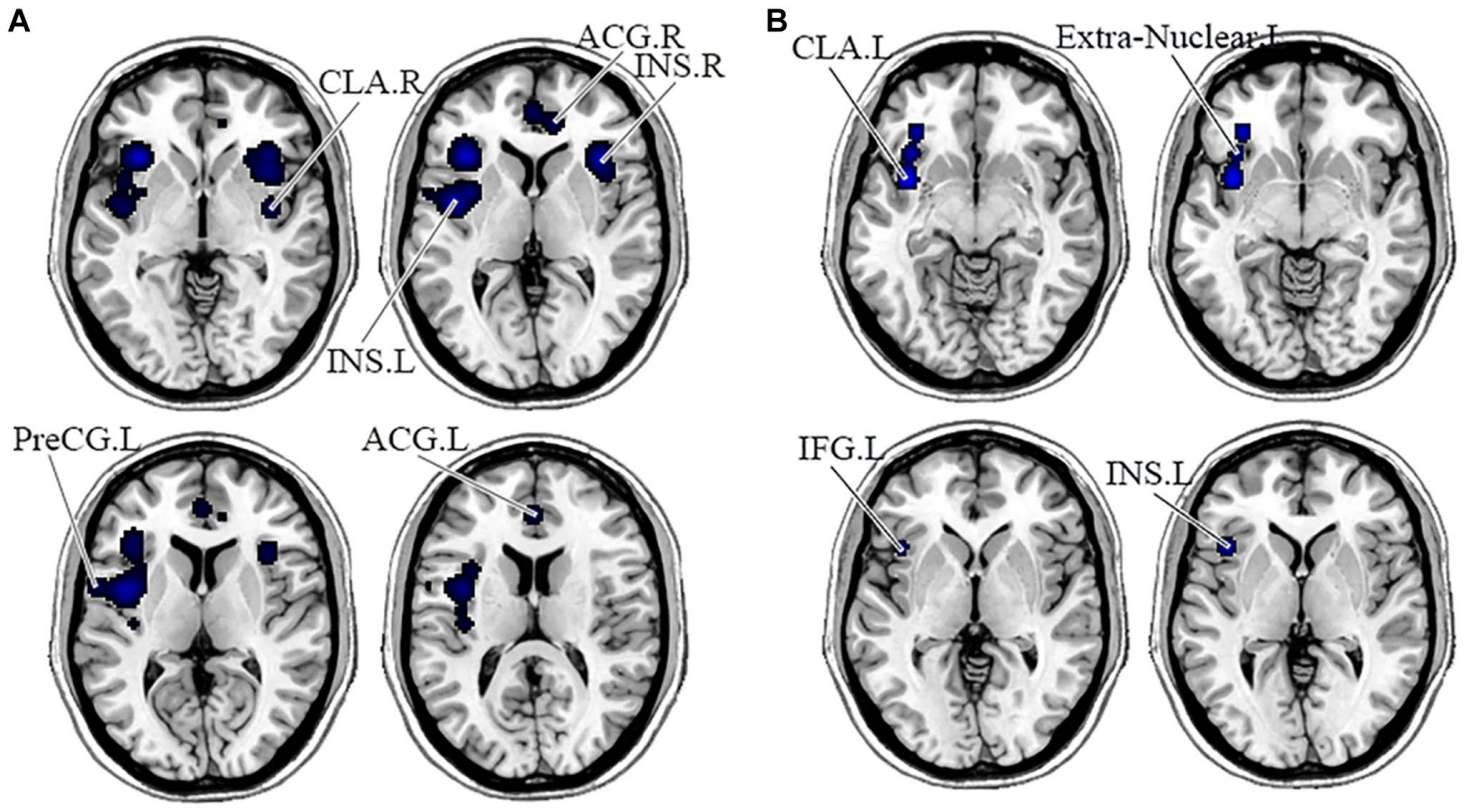
Displayed are significant results from the meta-analysis of the SN studies. **(A)** Gray matter reduction in the SN in PNS patients relative to HCs. **(B)** Areas showing lower FC in the SN in PNS patients relative to HCs. FC, functional connectivity; PNS, Persistent negative symptoms; HCs, healthy controls; SN, salience network; CLA, claustrum; ACG, anterior cingulate gyrus; INS, insula; PreCG, precentral gyrus; IFG, inferior frontal gyrus; R, right; L, left.

In the DMN, PNS patients showed decreased gray matter in the bilateral medial frontal gyrus (MFG) and left ACG and a significant reduction of FC in the right precuneus (Table 3 and Figure 2). Also, PNS patients had a decrease of gray matter in the left IPL and MFG and a significant reduction of FC in the bilateral SFG in the CEN (Table 3 and Figure 3). In comparison with HCs, PNS patients exhibited reduced gray matter in the bilateral insula, ACG, left precentral gyrus (PreCG) and right claustrum and lower FC in these brain areas in the SN, including the left insula, claustrum, inferior frontal gyrus (IFG) and extra-nuclear (Table 3 and Figure 4). No significantly increased FC was found in the patients in the three networks.

**Table 3:** Meta-analysis results for VBM and FC studies in patients with PNS and HCs.

Cluster	Volume (mm^3^)	MNI			Peak ALE value	Brain regions	Side	BA
		*x*	*y*	*z*				
Default mode network								
GMV:PNS < HC								
1	2,680	-6	46	-8	0.021635	Anterior cingulate	Left	32
1	2,680	8	50	14	0.017261	Medial frontal gyrus	Right	9
1	2,680	−6	54	2	0.010414839	Medial frontal gyrus	Left	10
FC: PNS < HC								
1	2,176	4	−62	38	0.017332	Precuneus	Right	7
Central executive network								
GMV: PNS < HC								
1	3,968	−58	−42	46	0.013759	Inferior parietal lobule	Left	40
1	3,968	−38	−40	56	0.008173	Inferior parietal lobule	Left	40
1	3,968	−50	−42	58	0.007295	Inferior parietal lobule	Left	40
2	2,288	−30	68	0	0.007809	Middle frontal gyrus	Left	10
2	2,288	−24	54	0	0.00768	Middle frontal gyrus	Left	10
FC: PNS < HC								
1	3,992	−24	60	18	0.011173	Superior frontal gyrus	Left	10
1	3,992	−20	54	34	0.009512	Superior frontal gyrus	Left	8
1	3,992	−18	60	32	0.008959	Superior frontal gyrus	Left	9
1	3,992	−26	52	38	0.006751	Superior frontal gyrus	Left	8
2	3,544	16	24	56	0.009713	Superior frontal gyrus	Right	6
2	3,544	26	30	50	0.009651	Superior frontal gyrus	Right	8
Salience network								
GMV: PNS < HC								
1	10,432	−36	22	0	0.027121	Insula	Left	13
1	10,432	−42	0	8	0.017298	Insula	Left	13
1	10,432	−44	−2	4	0.016858	Insula	Left	13
1	10,432	−58	2	8	0.008169	Precentral gyrus	Left	6
1	10,432	−44	10	−6	0.007955	Insula	Left	13
1	10,432	−34	12	12	0.006201	Insula	Left	13
1	10,432	−40	−12	18	0.005979	Insula	Left	13
1	10,432	−38	−18	12	0.005963	Insula	Left	13
2	6,672	36	22	2	0.019119	Insula	Right	13
2	6,672	34	14	−4	0.011924	Claustrum	Right	*
2	6,672	38	−6	−2	0.009962	Claustrum	Right	*
2	6,672	42	18	−10	0.008699	Insula	Right	47
2	6,672	42	4	−8	0.007584	Insula	Right	13
3	2,456	0	48	4	0.008874	Anterior cingulate	Left	32
3	2,456	10	42	4	0.007891	Anterior cingulate	Right	32
3	2,456	0	42	14	0.00782	Anterior cingulate	Left	32
FC: PNS < HC								
1	3,136	−38	6	−8	0.013396	Claustrum	Left	*
1	3,136	−34	32	−6	0.009221	Inferior frontal gyrus	Left	47
1	3,136	−36	20	−8	0.0081	Extra-nuclear	Left	47
1	3,136	−42	18	2	0.007982	Insula	Left	13

BA, Brodmann Area; ALE, Anatomical/Activation Likelihood Estimation; MNI, Montreal Neurologic Institute; PNS, Persistent negative symptoms; HC, healthy control; GMV, gray matter volume; FC, functional connectivity. *The peak coordinate is not at the Brodmann Area.

## 4. Discussion

This was the first meta-analysis to evaluate the functional and structural integrity of three brain networks in patients with PNS. Compared with the HCs, the PNS group had reduced GMV in bilateral MFG and left ACG, reduced FC in the right precuneus in the DMN; decreased GMV in the left IPL and MFG, and decreased FC in the bilateral SFG in the CEN; reduced GMV in the bilateral insula, ACG, left PreCG and right claustrum, and lower FC in the left insula, claustrum, IFG and extra-nuclear these in the SN. Similar to our results, an early report indicated that schizophrenia exhibited brain surface area contractions compared with normal controls in the three networks at the same time (Palaniyappan et al., 2011). Chronic and first-episode schizophrenia also had a significant reduction in the volume of gray matter (Chan et al., 2011; Koelkebeck et al., 2019; Sun et al., 2020), which relates to the severity of negative symptoms (Lei et al., 2019; Li et al., 2020). In addition, previous studies have shown altered FC of key regions in the DMN, CEN (Whitford et al., 2009; Repovs et al., 2011; Littow et al., 2015), and SN (Manoliu et al., 2013; Orliac et al., 2013; Wotruba et al., 2014). Overall, intra-network comparisons are useful to focus on recognizing brain areas with aberrant anatomical or functional alterations and therefore may be useful in detecting imaging features of PNS disease.

In our study, PNS patients showed structural and functional alterations in the DMN, including reduced GMV in bilateral MFG and left ACG, and reduced FC in the right precuneus. The MFG is located at the medial part of the prefrontal gyrus (Frascarelli et al., 2015), which is responsible for regulating emotional behavior and self-reference processes through the frontal-limbic circuit (Etkin et al., 2011; Chen et al., 2012; Müller et al., 2020). In many imaging studies, there is a consistent correlation between gray matter reduction in the prefrontal gyrus and the severity of negative symptoms (Cascella et al., 2010; Benoit et al., 2012; Jiang et al., 2019). The ACG, as a part of the ventromedial brain circuit, plays a critical role in evaluating the salience of emotional and motivational information and regulating emotional responses (Bush et al., 2000). Earlier findings indicated this region to be structurally altered and associated with negative symptoms (Sigmundsson et al., 2001; Baiano et al., 2007; Fornito et al., 2009; Berge et al., 2011), where the patients with the highest negative symptom levels show more gray matter loss (Asami et al., 2013). The precuneus modulates negative emotional responses primarily by triggering an attention shift in emotion regulation strategies (Ferri et al., 2016). Resting-state metabolic activities in the precuneus, dorsolateral prefrontal cortex and supplementary motor region had a negative correlation with physiological anhedonia in schizophrenia (Park et al., 2009). The pattern of functional interaction alterations in the DMN of schizophrenia patients has been universally correlated with negative symptoms (Park et al., 2009; Yang et al., 2019), indicating a key role for DMN in the etiology of negative symptoms and particularly in the field of apathy (Forlim et al., 2020). The primary function of the DMN includes self-referential processing (Buckner et al., 2008), so its abnormalities may result in disruptions in self-reflection and self-awareness, which may be the cause of anhedonia and apathy in schizophrenia.

Similarly, our study reported structural and functional alterations in the CEN, including decreased GMV in the left IPL and MFG, and reduced FC in the bilateral SFG. The gray matter loss in the IPL endorses the conception that IPL is engaged in the progression of schizophrenia with PNS (Sigmundsson et al., 2001). This may relate to the function of mirror neurons in the IPL in social cognition, especially empathy and understanding the behavior and intentions of others (Gallese et al., 2004; Rizzolatti and Sinigaglia, 2010). The prefrontal cortex, as a prototypical center of higher-order cognitive processing, is broadly connected to various brain regions (Meyer-Lindenberg et al., 2002; Barch and Dowd, 2010), and the interaction between synaptic plasticity and dopamine transmission in the prefrontal cortex has a prominent effect on psychotic symptoms in schizophrenia, particularly negative symptoms (Seamans and Yang, 2004). Numerous studies have reported the association between the prefrontal cortex and negative symptom severity, and rTMS treatment of the prefrontal cortex can reduce negative symptoms in schizophrenia (Brady et al., 2019; Kumar et al., 2020). The function of this region in negative symptoms requires further studies to elucidate.

Our study also reported reduced gray matter and lower FC in these brain areas in the SN, including the insula, PreCG, claustrum and extra-nuclear. Reduced GMV in the insula seems to be particularly associated with schizophrenia with PNS (Cascella et al., 2010; Li et al., 2018). The claustrum is a thin layer of neurons that appears to derive from migrating neuroblasts in the overlying cortex and lies below the insula and the temporal lobe (Galeno et al., 2004). Previous studies demonstrated a link between left insula dysfunction in schizophrenia and negative symptoms such as anhedonia and diminished social interactions (Manoliu et al., 2013), which might be related to impaired responses on pleasant stimuli caused by insula dysfunction. Despite few studies reporting the association between PreCG volume and negative symptoms, a prior meta-analysis found reduced GMV in the PreCG in schizophrenia with PNS (Li et al., 2018). The extra-nuclear region is located in the ventral emotional processing system and contains the main fibers connecting the striatum to the frontoparietal cortex, therefore structural or functional abnormalities of this pathway may affect emotional processing (Kring and Barch, 2014). Previous studies have reported that dysfunctioning of the cortico-striatal system has some connection with amotivation and anhedonia (Kring et al., 2013; Barnes et al., 2014). Early studies also indicated that in the triple-network model, SN-centered patients with low connectivity often had more severe and persistent negative symptoms than the subgroup of patients with high connectivity (Liang et al., 2021). However, we did not find that PNS patients had increased FC in the SN, which may be due to differences in patient samples, statistical methods and disease states. By summarizing these reports, our results confirm that PNS patients show more prominent GMV and FC decreases in these core networks than HCs, which may be the reason why patients with PNS exhibit a greater clinical symptom burden and poorer therapeutic outcomes.

In our study, the key region where DMN and CEN overlap is MFG, and the main region where DMN and SN overlap is ACG, suggesting that these networks interact in PNS patients. It is well known that the MFG and ACG play a key role in regulating emotional responses (Bush et al., 2000; Chen et al., 2012), and structural defects in these regions may be related to apathy in PNS patients. Moreover, self-rated avolition and anhedonia in schizophrenia are related to increased FC between the caudate and posterior DMN/CEN (Brakowski et al., 2020). It has been reported that connectivity between different subnetworks of CEN and DMN correlated with the severity of different clinical symptoms in schizophrenia (Xi et al., 2021). Taken together, one core network impairment affects other networks and its clinical aspects may surpass the initial disability. Significant impaired structure and function in the three networks signifies that schizophrenia with PNS involves multiple networks and the discovery of abnormalities in network connectivity has vital significance in searching for the network endophenotype of neuropsychiatric disease.

## 5. Limitations

This analysis has some limitations that should be considered. First, though we ascertained related studies by defining strict criteria for PNS, we were unable to ensure that negative symptoms persisted for at least 6 months and that all subjects included in the study met the PNS criteria, which may led to the heterogeneity of results. Second, the brain region of the three networks selected for the included studies may be influenced by the *a priori* hypotheses of the authors of each study. The clusters detected in our meta-analysis were facilitated by minority studies and require a greater sample size in the future. Third, the ALE software could not investigate the effect size differences between networks, and it failed to provide any solving approach to analyze the confidence interval to increase the robustness of our findings. Fourth, the study was limited by heterogeneity, including different data sources, different negative symptom assessment scales, preprocessing protocols, smoothing kernel size, slice thickness, and statistical threshold, which may have influenced our results in this study. Lastly, there is a great deal of heterogeneity among schizophrenia patients, including age of onset and dose of antipsychotic medications.

## 6. Conclusion and perspectives

This meta-analysis illuminates within-network comparisons to assist in the key identification of brain areas with abnormal anatomical or functional alterations within the DMN, CEN, and SN. The meaningful overlap of brain regions between three networks opens up new ideas for selecting specific brain regions as therapeutic targets for rTMS in the future. These current findings extend our understanding of patients with PNS through a brain network-level perspective and provide a starting point for designing targeted and effective interventions. However, the study of imaging markers in PNS patients is still in the preliminary stage, and the limitations of insufficient study samples and single study methods lead to the lack of consistent conclusions. In the future, neuroimaging studies should combine molecular biology, genomics, epigenetics, etc., to explore the formation mechanism of PNS at multiple levels, use deep learning and other methods to explore stable and reliable imaging markers of PNS patients and combine multi-modal biological indicators to explore biological targets conducive to diagnosis and curative effect prediction.

## Author contributions

TZ guided by XZ and JC designed the study. ZeW, WW, and YL performed the meta-analysis and drafted the manuscript. ZiW, CZ, XF, CH, and CX helped in literature extraction and data analyses. All authors contributed to and approved the final text.

## Footnotes

^1^http://brainmap.org/ale/

^2^http://rfmri.org/dpabi
