# Supplementary material for: Altered brain functional networks in schizophrenia with persistent negative symptoms: an activation likelihood estimation meta-analysis
Source: Front Hum Neurosci. 2023 Oct 26;17:1204632. doi: 10.3389/fnhum.2023.1204632 (PMC10637389; doi:10.3389/fnhum.2023.1204632)
Supplement: Supplementary file 1 [file Table_1.docx]

Table S1 Characteristics of studies of VBM

| study | SZ | | HC | | NS scale | NS | PS | DOI | Brain regions |
| --- | --- | --- | --- | --- | --- | --- | --- | --- | --- |
|  | No. | Age | No. | Age |  |  |  |  |  |
| ***Default Mode Network*** |  |  |  |  |  |  |  |  |  |
| Neugebauer et al. (2019)[1] | 18 | 36.94 | 19 | 35.79 | PANSS | 23.61 | 15.33 | 12.58 | PCUN.R; PCUN.L; MFG.L |
| Spalthoff et al. (2018)[2] | 51 | 35.18 | 102 | 33.15 | SANS | 42.45 | 19.38 | 8.8 | IPL.R |
| Szendi et al. (2017)[3] | 8 | 34 | 13 | 34 | PANSS | 27.5 | 17.5 | 13 | mOFG.R |
| Kuroki et al. (2017)[4] | 15 | 44.1 | 23 | 36.8 | PANSS | 19.7 | 14.3 | 18 | PCUN.R; PCUN.L; PCG.L |
| Poletti et al. (2016)[5] | 96 | 37.24 | 136 | 33.31 | PANSS | 20.41 | 17.62 | 12.61 | MFG |
| Huang et al. (2015)[6] | 18 | 22.67 | 18 | 25.06 | PANSS | 22.06 | 18.61 | 1.04 | IPL.L;MTG.R;mOFG.R |
| Whitford et al. (2009)[7] | 31 | 19.3 | 21 | 19.6 | PANSS | 20 | 18 | 0.53 | MFG |
| Herold et al. (2009)[8] | 18 | 28.7 | 21 | 27.4 | PANSS | 19.6 | 14.2 | 3.4 | MTG;ITG mOFG.L |
| Meisenzahl et al. (2008)[9] | 93 | 28.2 | 177 | 31.5 | PANSS | 20.4 | 19.9 | 0.76 | mOFG.L;MFG.L;ITG.L;MTG.L |
| Koutsouleris et al. (2008)[10] | 175 | 31.7 | 177 | 31.5 | PANSS | 22.3 | 19 | 1.6 | IPL.L;MTG.R;mOFG.L;MFG.R;PCUN.L;PCUN.R |
| Bassitt et al. (2007)[11] | 50 | 31.7 | 30 | 31.2 | PANSS | 19.8 | 12.9 | 11.4 | PCUN.R |
| Jayakumar et al. (2005)[12] | 18 | 24.9 | 18 | 25.7 | PANSS | 23 | 19 | 0.86 | IPL |
| Salgado-Pineda et al. (2004)[13] | 14 | 25.05 | 14 | 25.14 | SANS | 21.21 | 9.78 | 1 | MFG;IPL.R;ITG |
| Sigmundsson et al. (2001)[14] | 27 | 34.9 | 27 | 32.2 | PANSS | 25 | 14.7 | 13.9 | MFG |
| Paillère-Martinot et al. (2001)[15] | 20 | 29 | 20 | 26 | PANSS | 27.6 | 17.3 | 10 | MFG |
| ***Central Executive Network*** |  |  |  |  |  |  |  |  |  |
| Herold et al. (2009)[8] | 18 | 28.7 | 21 | 27.4 | PANSS | 19.6 | 14.2 | 3.4 | SFG |
| Meisenzahl et al. (2008)[9] | 93 | 28.2 | 177 | 31.5 | PANSS | 20.4 | 19.9 | 0.76 | SFG |
| Koutsouleris et al. (2008)[10] | 175 | 31.7 | 177 | 31.5 | PANSS | 22.3 | 19 | 1.6 | SFG |
| Bassitt et al. (2007)[11] | 50 | 31.7 | 30 | 31.2 | PANSS | 19.8 | 12.9 | 11.4 | dlPFG |
| Jayakumar et al. (2005)[12] | 18 | 24.9 | 18 | 25.7 | PANSS | 23 | 19 | 0.86 | SFG |
| ***Salience Network*** |  |  |  |  |  |  |  |  |  |
| Neugebauer et al. (2019)[1] | 18 | 36.94 | 19 | 35.79 | PANSS | 23.61 | 15.33 | 12.6 | INS;ACG.R |
| Spalthoff et al. (2018)[2] | 51 | 35.18 | 102 | 33.15 | SANS | 42.45 | 19.38 | 8.8 | INS.R |
| Szendi et al. (2017)[3] | 8 | 34 | 13 | 34 | PANSS | 27.5 | 17.5 | 13 | INS |
| Kuroki et al. (2017)[4] | 15 | 44.1 | 23 | 36.8 | PANSS | 19.7 | 14.3 | 18 | INS |
| Kim et al. (2017)[16] | 22 | 31.7 | 22 | 31.6 | PANSS | 21.1 | 18.4 | 9.2 | INS;ACG |
| Anderson et al. (2015)[17] | 15 | 34.3 | 20 | 33.3 | PANSS | 20 | 13 | 11.4 | ACG |
| Poletti et al. (2016)[5] | 96 | 37.24 | 136 | 33.31 | PANSS | 20.41 | 17.62 | 12.6 | INS |
| Herold et al. (2009)[8] | 18 | 28.7 | 21 | 27.4 | PANSS | 19.6 | 14.2 | 3.4 | INS.L |
| Meisenzahl et al. (2008)[9] | 93 | 28.2 | 177 | 31.5 | PANSS | 20.4 | 19.9 | 0.76 | INS.L |
| Koutsouleris et al. (2008)[10] | 175 | 31.7 | 177 | 31.5 | PANSS | 22.3 | 19 | 1.6 | INS |
| Bassitt et al. (2007)[11] | 50 | 31.7 | 30 | 31.2 | PANSS | 19.8 | 12.9 | 11.4 | INS |
| Jayakumar et al. (2005)[12] | 18 | 24.9 | 18 | 25.7 | PANSS | 23 | 19 | 0.86 | INS.L |
| Salgado-Pineda et al. (2004)[13] | 14 | 25.05 | 14 | 25.14 | SANS | 21.21 | 9.78 | 1 | ACG.R |
| Sigmundsson et al. (2001)[14] | 27 | 34.9 | 27 | 32.2 | PANSS | 25 | 14.7 | 13.9 | ACG |
| Paillère-Martinot et al. (2001)[15] | 20 | 29 | 20 | 26 | PANSS | 27.6 | 17.3 | 10 | INS.L |

SZ, schizophrenia; HC, healthy control; VBM, voxel-based morphometry; DOI, duration of illness; M/F, male/female; PANSS, Positive and Negative Syndrome Scale; SANS, Scale for the Assessment of Negative Symptom; NS, negative symptoms; PS: positive symptoms; PCUN, precuneus; MFG, medial frontal gyrus; IPL, inferior parietal lobule; mOFC, medial orbital frontal gyrus; MTG, medial temporal gyrus; ITG, inferior temporal gyrus; SFG, superior frontal gyrus; dlPFG, dorsolateral prefrontal gyrus; INS, insula; ACG, anterior cingulate gyrus; R, right; L, left.

Table S2 Characteristics of studies of FC

| study | SZ |  | HC |  | NS scale | NS | PS | DOI | Brain regions |
| --- | --- | --- | --- | --- | --- | --- | --- | --- | --- |
|  | No. | Age | No. | Age |  |  |  |  |  |
| ***Default Mode Network*** |  |  |  |  |  |  |  |  |  |
| Dong et al. (2019)[18] | 96 | 39.8 | 122 | 38.0 | PANSS | 20.73 | 13.44 | 15.1 | PCG;MTG;MFG;IPL |
| Sharma et al. (2018)[19] | 34 | 29.32 | 19 | 31.53 | SANS | 31.74 | 15 | 3.73 | PCG.R;MTG.L |
| Penner et al. (2018a)[20] | 24 | 23.2 | 24 | 23.8 | SANS | 22.5 | 10.3 | 13.7 | IPL.L;dlPFC.R;PCUN.R; PCG.R/L |
| Penner et al. (2018b)[21] | 24 | 23.2 | 24 | 23.8 | SANS | 22.5 | 10.3 | 13.7 | PCUN.L;MFG.R/L |
| Liu et al. (2018)[22] | 21 | 30.95 | 21 | 31.43 | PANSS | 22.19 | 11.62 | 4.74 | MFG |
| C. J. Zhuo et al. (2017)[23] | 95 | 33.6 | 93 | 33 | PANSS | 20.3 | 17.1 | 10.12 | IPL.L; |
| Peters et al. (2017)[24] | 21 | 34.05 | 21 | 33.49 | PANSS | 21.14 | 19.4 | 7.15 | MFG |
| H. L. Wang et al. (2016)[25] | 31 | 20.61 | 37 | 20.76 | PANSS | 20.32 | 20 | 0.43 | PCG.R/L;MFG.L |
| Penner et al. (2016)[26] | 24 | 23.2 | 24 | 23.8 | SANS | 22.5 | 10.3 | 1.14 | MTG. R/L;MFG. R/L; PCUN. R/L |
| Chen et al. (2016)[27] | 46 | 41.54 | 46 | 39.05 | PANSS | 20.61 | 12.52 | 16.27 | IPL. R/L |
| Zhou et al. (2015)[28] | 91 | 33.8 | 100 | 33.3 | PANSS | 20 | 16.6 | 10 | MFG.L;PCUN.L |
| Xu et al. (2015)[29] | 66 | 33 | 76 | 33 | PANSS | 21.1 | 17 | 9.5 | PCG.L |
| D. Wang et al. (2015)[30] | 94 | 33.6 | 102 | 33.4 | PANSS | 20.3 | 16.6 | 10 | PCG |
| Alonso-Solís et al. (2015)[31] | 19 | 40.05 | 20 | 37.75 | PANSS | 21.47 | 17.89 | 16.11 | MFG;PCG;IPL;MTG |
| C. Zhuo et al. (2014)[32] | 95 | 33.6 | 93 | 33 | PANSS | 20.3 | 17.1 | 10.12 | MTG.R |
| Chang et al. (2014)[33] | 25 | 25.36 | 25 | 25.48 | PANSS | 21.39 | 18.73 | 1.53 | IPL.R;PCUN |
| Manoliu et al. (2014)[34] | 18 | 35.33 | 20 | 34 | PANSS | 19.94 | 18.06 | 7 | PCUN;MFG.L;IPL; |
| Fan et al. (2013)[35] | 27 | 39.7 | 15 | 41.4 | PANSS | 20.9 | 18.9 | 16.5 | MFG;MTG |
| Wolf et al. (2011)[36] | 10 | 36.5 | 14 | 33.7 | PANSS | 22 | 16 | 9.9 | PCG.R;PCUN.L |
| Bluhm et al. (2007)[37] | 17 | 33.54 | 17 | 30.94 | SANS | 20.35 | 9.06 | 9.78 | MFG;PCUN.R |
| ***Central Executive Network*** |  |  |  |  |  |  |  |  |  |
| Penner et al. (2018a)[20] | 24 | 23.2 | 24 | 23.8 | SANS | 22.5 | 10.3 | 13.7 | SFG |
| Penner et al.(2018b）[21] | 24 | 23.2 | 24 | 23.8 | SANS | 22.5 | 10.3 | 13.7 | dlPFG.R |
| Peters et al. (2017)[24] | 21 | 34.05 | 21 | 33.49 | PANSS | 21.14 | 19.4 | 7.15 | SFG.R |
| Penner et al. (2016)[26] | 24 | 23.2 | 24 | 23.8 | SANS | 22.5 | 10.3 | 1.14 | SFG |
| Zhou et al. (2015)[28] | 91 | 33.8 | 100 | 33.3 | PANSS | 20 | 16.6 | 10 | SFG |
| Chang et al. (2014)[33] | 25 | 25.36 | 25 | 25.48 | PANSS | 21.39 | 18.73 | 1.53 | SFG.L |
| Manoliu et al. (2014)[34] | 18 | 35.33 | 20 | 34 | PANSS | 19.94 | 18.06 | 7 | SFG |
| ***Salience Network*** |  |  |  |  |  |  |  |  |  |
| Penner et al. (2018a)[20] | 24 | 23.2 | 24 | 23.8 | SANS | 22.5 | 10.3 | 13.7 | INS.L |
| Penner et al.(2018b）[21] | 24 | 23.2 | 24 | 23.8 | SANS | 22.5 | 10.3 | 13.7 | ACG.L |
| Peters et al. (2017)[24] | 21 | 34.05 | 21 | 33.49 | PANSS | 21.14 | 19.4 | 7.15 | INS |
| Penner et al. (2016)[26] | 24 | 23.2 | 24 | 23.8 | SANS | 22.5 | 10.3 | 1.14 | INS;ACG |
| Chen et al. (2016)[27] | 46 | 41.54 | 46 | 39.05 | PANSS | 20.61 | 12.52 | 16.27 | INS |
| Zhou et al. (2015)[28] | 91 | 33.8 | 100 | 33.3 | PANSS | 20 | 16.6 | 10 | ACG.L |
| Xu et al. (2015)[29] | 66 | 33 | 76 | 33 | PANSS | 21.1 | 17 | 9.5 | INS.R |
| D. Wang et al. (2015)[30] | 94 | 33.6 | 102 | 33.4 | PANSS | 20.3 | 16.6 | 10 | ACG |
| Manoliu et al. (2014)[34] | 18 | 35.33 | 20 | 34 | PANSS | 19.94 | 18.06 | 7 | ACG;INS |
| Wolf et al. (2011)[36] | 10 | 36.5 | 14 | 33.7 | PANSS | 22 | 16 | 9.9 | ACG.L |

SZ, schizophrenia; HC, healthy control; FC, functional connectivity; DOI, duration of illness; M/F, male/female; PANSS, Positive and Negative Syndrome Scale; SANS, Scale for the Assessment of Negative Symptom; SDS, Schedule of Deficit Syndrome; NS, negative symptoms; PS: positive symptoms; PCUN, precuneus; MFG, medial frontal gyrus; IPL, inferior parietal lobule; mOFC, medial orbital frontal gyrus; MTG, medial temporal gyrus; SFG, superior frontal gyrus; dlPFG, dorsolateral prefrontal gyrus; INS, insula; ACG, anterior cingulate gyrus; R, right; L, left.

1. Neugebauer, K., et al., *Nerve Growth Factor Serum Levels Are Associated With Regional Gray Matter Volume Differences in Schizophrenia Patients.* Front Psychiatry, 2019. **10**: p. 275.

2. Spalthoff, R., C. Gaser, and I. Nenadić, *Altered gyrification in schizophrenia and its relation to other morphometric markers.* Schizophr Res, 2018. **202**: p. 195-202.

3. Szendi, I., et al., *A New Division of Schizophrenia Revealed Expanded Bilateral Brain Structural Abnormalities of the Association Cortices.* Front Psychiatry, 2017. **8**: p. 127.

4. Kuroki, N., et al., *Brain structure differences among male schizophrenic patients with history of serious violent acts: an MRI voxel-based morphometric study.* BMC Psychiatry, 2017. **17**(1): p. 105.

5. Poletti, S., et al., *Adverse childhood experiences influence the detrimental effect of bipolar disorder and schizophrenia on cortico-limbic grey matter volumes.* J Affect Disord, 2016. **189**: p. 290-7.

6. Huang, P., et al., *Decreased bilateral thalamic gray matter volume in first-episode schizophrenia with prominent hallucinatory symptoms: A volumetric MRI study.* Sci Rep, 2015. **5**: p. 14505.

7. Whitford, T.J., et al., *Delusions and dorso-medial frontal cortex volume in first-episode schizophrenia: a voxel-based morphometry study.* Psychiatry Res, 2009. **172**(3): p. 175-9.

8. Herold, R., et al., *Regional gray matter reduction and theory of mind deficit in the early phase of schizophrenia: a voxel-based morphometric study.* Acta Psychiatr Scand, 2009. **119**(3): p. 199-208.

9. Meisenzahl, E.M., et al., *Structural brain alterations at different stages of schizophrenia: A voxel-based morphometric study.* Schizophrenia Research, 2008. **104**(1-3): p. 44-60.

10. Koutsouleris, N., et al., *Structural correlates of psychopathological symptom dimensions in schizophrenia: a voxel-based morphometric study.* Neuroimage, 2008. **39**(4): p. 1600-12.

11. Bassitt, D.P., et al., *Insight and regional brain volumes in schizophrenia.* Eur Arch Psychiatry Clin Neurosci, 2007. **257**(1): p. 58-62.

12. Jayakumar, P.N., et al., *Optimized voxel-based morphometry of gray matter volume in first-episode, antipsychotic-naive schizophrenia.* Prog Neuropsychopharmacol Biol Psychiatry, 2005. **29**(4): p. 587-91.

13. Salgado-Pineda, P., et al., *Decreased cerebral activation during CPT performance: structural and functional deficits in schizophrenic patients.* Neuroimage, 2004. **21**(3): p. 840-7.

14. Sigmundsson, T., et al., *Structural abnormalities in frontal, temporal, and limbic regions and interconnecting white matter tracts in schizophrenic patients with prominent negative symptoms.* Am J Psychiatry, 2001. **158**(2): p. 234-43.

15. Paillère-Martinot, M., et al., *Cerebral gray and white matter reductions and clinical correlates in patients with early onset schizophrenia.* Schizophr Res, 2001. **50**(1-2): p. 19-26.

16. Kim, G.W., Y.H. Kim, and G.W. Jeong, *Whole brain volume changes and its correlation with clinical symptom severity in patients with schizophrenia: A DARTEL-based VBM study.* PLoS One, 2017. **12**(5): p. e0177251.

17. Anderson, V.M., et al., *Extensive Gray Matter Volume Reduction in Treatment-Resistant Schizophrenia.* International Journal of Neuropsychopharmacology, 2015. **18**(7).

18. Dong, D.B., et al., *Reconfiguration of Dynamic Functional Connectivity in Sensory and Perceptual System in Schizophrenia.* Cerebral Cortex, 2019. **29**(8): p. 3577-3589.

19. Sharma, A., et al., *Altered resting state functional connectivity in early course schizophrenia.* Psychiatry Res Neuroimaging, 2018. **271**: p. 17-23.

20. Penner, J., et al., *Higher order thalamic nuclei resting network connectivity in early schizophrenia and major depressive disorder.* Psychiatry Res Neuroimaging, 2018. **272**: p. 7-16.

21. Penner, J., et al., *Temporoparietal Junction Functional Connectivity in Early Schizophrenia and Major Depressive Disorder.* Chronic Stress (Thousand Oaks), 2018. **2**: p. 2470547018815232.

22. Liu, H., et al., *Cigarette smoking and schizophrenia independently and reversibly altered intrinsic brain activity.* Brain Imaging Behav, 2018. **12**(5): p. 1457-1465.

23. Zhuo, C.J., et al., *Brain structural and functional dissociated patterns in schizophrenia.* Bmc Psychiatry, 2017. **17**.

24. Peters, H., et al., *Changes in extra-striatal functional connectivity in patients with schizophrenia in a psychotic episode.* British Journal of Psychiatry, 2017. **210**(1): p. 75-82.

25. Wang, H.L., et al., *Patients with first-episode, drug-naive schizophrenia and subjects at ultra-high risk of psychosis shared increased cerebellar-default mode network connectivity at rest.* Scientific Reports, 2016. **6**.

26. Penner, J., et al., *Medial Prefrontal and Anterior Insular Connectivity in Early Schizophrenia and Major Depressive Disorder: A Resting Functional MRI Evaluation of Large-Scale Brain Network Models.* Front Hum Neurosci, 2016. **10**: p. 132.

27. Chen, X., et al., *Functional abnormalities of the right posterior insula are related to the altered self-experience in schizophrenia.* Psychiatry Research - Neuroimaging, 2016. **256**: p. 26-32.

28. Zhou, Y., et al., *The selective impairment of resting-state functional connectivity of the lateral subregion of the frontal pole in schizophrenia.* PLoS One, 2015. **10**(3): p. e0119176.

29. Xu, L., et al., *Selective Functional Disconnection of the Dorsal Subregion of the Temporal Pole in Schizophrenia.* Sci Rep, 2015. **5**: p. 11258.

30. Wang, D., et al., *Altered functional connectivity of the cingulate subregions in schizophrenia.* Transl Psychiatry, 2015. **5**(6): p. e575.

31. Alonso-Solís, A., et al., *Resting-state functional connectivity alterations in the default network of schizophrenia patients with persistent auditory verbal hallucinations.* Schizophr Res, 2015. **161**(2-3): p. 261-8.

32. Zhuo, C., et al., *Functional connectivity density alterations in schizophrenia.* Front Behav Neurosci, 2014. **8**: p. 404.

33. Chang, X., et al., *Altered default mode and fronto-parietal network subsystems in patients with schizophrenia and their unaffected siblings.* Brain Res, 2014. **1562**: p. 87-99.

34. Manoliu, A., et al., *Aberrant dependence of default mode/central executive network interactions on anterior insular salience network activity in schizophrenia.* Schizophr Bull, 2014. **40**(2): p. 428-37.

35. Fan, F.M., et al., *Ventral medial prefrontal functional connectivity and emotion regulation in chronic schizophrenia: a pilot study.* Neurosci Bull, 2013. **29**(1): p. 59-74.

36. Wolf, N.D., et al., *Dysconnectivity of multiple resting-state networks in patients with schizophrenia who have persistent auditory verbal hallucinations.* Journal of Psychiatry & Neuroscience, 2011. **36**(6): p. 366-374.

37. Bluhm, R.L., et al., *Spontaneous low-frequency fluctuations in the BOLD signal in schizophrenic patients: anomalies in the default network.* Schizophr Bull, 2007. **33**(4): p. 1004-12.
