# Supplementary material for: Altered brain functional networks in schizophrenia with persistent negative symptoms: an activation likelihood estimation meta-analysis
Source: Front Hum Neurosci. 2023 Oct 26;17:1204632. doi: 10.3389/fnhum.2023.1204632 (PMC10637389; doi:10.3389/fnhum.2023.1204632)
Supplement: Supplementary file 2 [file Table_2.docx]

**Table S2. The checklist of imaging methodology quality assessment for all the articles included in the present meta-analysis.**

| **Study** | **Category 1: Subjects** | | | | **Category 2: Methods for image acquisition and analysis** | | | | | | **Category 3: Results and conclusions** | |  |
| --- | --- | --- | --- | --- | --- | --- | --- | --- | --- | --- | --- | --- | --- |
|  | Patients were evaluated prospectively, specific diagnostic criteria were applied, and demographic data were reported | Healthy comparison participants were evaluated prospectively; psychiatric and medical illnesses were excluded | Important variables (e.g., age, gender, drug status, illness duration, and symptom severity) were checked either via stratification or statistics | Sample size per group: ≥ 20, scores 1; ≥ 10, scores 0.5 | All neuroanatomic measurements were made blind to group assignment and to subjects’ identity | Measures for brain structures were reported | Magnet strength: 3T, scores 1; 1.5T, scores 0.5 | The imaging technique used was clearly described so that it could be reproduced | Whole brain analysis was automated without a previously defined region | Spatial coordinates were reported in a standard space (e.g., Talairach or MNI coordinates) | Statistical results were corrected for multiple comparison scores 1, uncorrected scores 0.5 | Conclusions were consistent with the results obtained, and the limitations were discussed | total |
| **GMV** | | | | | | | | | | | | | |
| Neugebauer^[1]^ | 1 | 1 | 1 | 0.5 | 1 | 1 | 1 | 1 | 1 | 1 | 1 | 1 | 11.5 |
| Spalthoff^[2]^ | 1 | 1 | 1 | 1 | 1 | 1 | 1 | 1 | 1 | 1 | 1 | 1 | 12 |
| Szendi^[3]^ | 1 | 1 | 1 | 0.5 | 1 | 1 | 0.5 | 1 | 1 | 1 | 1 | 1 | 11 |
| Kuroki^[4]^ | 1 | 1 | 0.5 | 0.5 | 1 | 1 | 1 | 1 | 1 | 1 | 1 | 1 | 11 |
| Kim^[5]^ | 1 | 1 | 1 | 1 | 1 | 1 | 1 | 1 | 1 | 1 | 1 | 1 | 12 |
| Poletti^[6]^ | 1 | 1 | 0.5 | 1 | 1 | 1 | 1 | 1 | 1 | 1 | 1 | 1 | 11.5 |
| Huang,P^[7]^ | 1 | 1 | 0.5 | 0.5 | 1 | 1 | 1 | 1 | 1 | 1 | 0.5 | 1 | 10.5 |
| Anderson^[8]^ | 1 | 1 | 0.5 | 0.5 | 1 | 1 | 1 | 1 | 1 | 1 | 1 | 1 | 11 |
| Whitford^[9]^ | 1 | 1 | 0.5 | 1 | 1 | 1 | 0.5 | 1 | 1 | 1 | 1 | 1 | 11 |
| Herold^[10]^ | 1 | 1 | 1 | 0.5 | 1 | 1 | 0.5 | 1 | 1 | 1 | 0.5 | 1 | 10.5 |
| Meisenzahl^[11]^ | 1 | 1 | 0.5 | 1 | 1 | 1 | 0.5 | 1 | 1 | 1 | 1 | 1 | 11 |
| Koutsouleris^[12]^ | 1 | 1 | 0.5 | 1 | 1 | 1 | 0.5 | 1 | 1 | 1 | 1 | 1 | 11 |
| Bassitt^[13]^ | 1 | 1 | 0.5 | 1 | 1 | 1 | 0.5 | 1 | 1 | 1 | 1 | 1 | 11 |
| Jayakumar^[14]^ | 1 | 1 | 1 | 0.5 | 1 | 1 | 0.5 | 1 | 1 | 1 | 1 | 1 | 11 |
| Salgado-Pineda^[15]^ | 1 | 1 | 0.5 | 0.5 | 1 | 1 | 0.5 | 1 | 1 | 1 | 1 | 1 | 10.5 |
| Sigmundsson^[16]^ | 1 | 1 | 0.5 | 1 | 1 | 1 | 0.5 | 1 | 1 | 1 | 0.5 | 1 | 10.5 |
| Paillère-Martinot^[17]^ | 1 | 1 | 1 | 1 | 1 | 1 | 0.5 | 1 | 1 | 1 | 0.5 | 1 | 11 |
| **FC** | | | | | | | | | | | | | |
| Dong^[18]^ | 1 | 1 | 1 | 1 | 1 | 1 | 1 | 1 | 1 | 1 | 1 | 1 | 12 |
| Sharma^[19]^ | 1 | 1 | 0.5 | 1 | 1 | 1 | 1 | 1 | 1 | 1 | 1 | 1 | 11.5 |
| Penner,J.a^[20]^ | 1 | 1 | 0.5 | 1 | 1 | 1 | 1 | 1 | 1 | 1 | 1 | 1 | 11.5 |
| Penner,J.b^[21]^ | 1 | 1 | 0.5 | 1 | 1 | 1 | 1 | 1 | 1 | 1 | 1 | 1 | 11.5 |
| Liu,H^[22]^ | 1 | 1 | 1 | 1 | 1 | 1 | 1 | 1 | 1 | 1 | 1 | 1 | 12 |
| Zhuo,C^[23]^ | 1 | 1 | 0.5 | 1 | 1 | 1 | 1 | 1 | 1 | 1 | 1 | 1 | 11.5 |
| Peters^[24]^ | 1 | 1 | 0.5 | 1 | 1 | 1 | 1 | 1 | 1 | 1 | 1 | 1 | 11.5 |
| Wang,H^[25]^ | 1 | 1 | 1 | 1 | 1 | 1 | 1 | 1 | 1 | 1 | 1 | 1 | 12 |
| Penner,J^[26]^ | 1 | 1 | 1 | 1 | 1 | 1 | 1 | 1 | 1 | 1 | 1 | 1 | 12 |
| Chen,X^[27]^ | 1 | 1 | 1 | 1 | 1 | 1 | 1 | 1 | 1 | 1 | 1 | 1 | 12 |
| Zhou,Y^[28]^ | 1 | 1 | 0.5 | 1 | 1 | 1 | 1 | 1 | 1 | 1 | 1 | 1 | 11.5 |
| Xu,L^[29]^ | 1 | 1 | 0.5 | 1 | 1 | 1 | 1 | 1 | 1 | 1 | 1 | 1 | 11.5 |
| Wang,D^[30]^ | 1 | 1 | 0.5 | 1 | 1 | 1 | 1 | 1 | 1 | 1 | 1 | 1 | 11.5 |
| Alonso-Solís^[31]^ | 1 | 1 | 0.5 | 0.5 | 1 | 1 | 1 | 1 | 1 | 1 | 1 | 1 | 11 |
| Zhuo,C^[32]^ | 1 | 1 | 0.5 | 1 | 1 | 1 | 1 | 1 | 1 | 1 | 1 | 1 | 11.5 |
| Manoliu^[33]^ | 1 | 1 | 0.5 | 0.5 | 1 | 1 | 1 | 1 | 1 | 1 | 0.5 | 1 | 10.5 |
| Chang,X^[34]^ | 1 | 1 | 1 | 1 | 1 | 1 | 1 | 1 | 1 | 1 | 1 | 1 | 12 |
| Fan,F.M^[35]^ | 1 | 1 | 0.5 | 1 | 1 | 1 | 1 | 1 | 1 | 1 | 1 | 1 | 11.5 |
| Wolf,N^[36]^ | 1 | 1 | 1 | 0.5 | 1 | 1 | 1 | 1 | 1 | 1 | 0.5 | 1 | 11 |
| Bluhm^[37]^ | 1 | 1 | 0.5 | 0.5 | 1 | 1 | 1 | 1 | 1 | 1 | 0.5 | 1 | 10.5 |

1. Neugebauer, K., et al., Nerve Growth Factor Serum Levels Are Associated With Regional Gray Matter Volume Differences in Schizophrenia Patients. Front Psychiatry, 2019. 10: p. 275.

2. Spalthoff, R., C. Gaser, and I. Nenadić, Altered gyrification in schizophrenia and its relation to other morphometric markers. Schizophr Res, 2018. 202: p. 195-202.

3. Szendi, I., et al., A New Division of Schizophrenia Revealed Expanded Bilateral Brain Structural Abnormalities of the Association Cortices. Front Psychiatry, 2017. 8: p. 127.

4. Kuroki, N., et al., Brain structure differences among male schizophrenic patients with history of serious violent acts: an MRI voxel-based morphometric study. BMC Psychiatry, 2017. 17(1): p. 105.

5. Kim, G.W., Y.H. Kim, and G.W. Jeong, Whole brain volume changes and its correlation with clinical symptom severity in patients with schizophrenia: A DARTEL-based VBM study. PLoS One, 2017. 12(5): p. e0177251.

6. Poletti, S., et al., Adverse childhood experiences influence the detrimental effect of bipolar disorder and schizophrenia on cortico-limbic grey matter volumes. J Affect Disord, 2016. 189: p. 290-7.

7. Huang, P., et al., Decreased bilateral thalamic gray matter volume in first-episode schizophrenia with prominent hallucinatory symptoms: A volumetric MRI study. Sci Rep, 2015. 5: p. 14505.

8. Anderson, V.M., et al., Extensive Gray Matter Volume Reduction in Treatment-Resistant Schizophrenia. International Journal of Neuropsychopharmacology, 2015. 18(7).

9. Whitford, T.J., et al., Delusions and dorso-medial frontal cortex volume in first-episode schizophrenia: A voxel-based morphometry study. Psychiatry Research-Neuroimaging, 2009. 172(3): p. 175-179.

10. Herold, R., et al., Regional gray matter reduction and theory of mind deficit in the early phase of schizophrenia: a voxel-based morphometric study. Acta Psychiatr Scand, 2009. 119(3): p. 199-208.

11. Meisenzahl, E.M., et al., Structural brain alterations at different stages of schizophrenia: A voxel-based morphometric study. Schizophrenia Research, 2008. 104(1-3): p. 44-60.

12. Koutsouleris, N., et al., Structural correlates of psychopathological symptom dimensions in schizophrenia: a voxel-based morphometric study. Neuroimage, 2008. 39(4): p. 1600-12.

13. Bassitt, D.P., et al., Insight and regional brain volumes in schizophrenia. Eur Arch Psychiatry Clin Neurosci, 2007. 257(1): p. 58-62.

14. Jayakumar, P.N., et al., Optimized voxel-based morphometry of gray matter volume in first-episode, antipsychotic-naive schizophrenia. Prog Neuropsychopharmacol Biol Psychiatry, 2005. 29(4): p. 587-91.

15. Salgado-Pineda, P., et al., Decreased cerebral activation during CPT performance: structural and functional deficits in schizophrenic patients. Neuroimage, 2004. 21(3): p. 840-7.

16. Sigmundsson, T., et al., Structural abnormalities in frontal, temporal, and limbic regions and interconnecting white matter tracts in schizophrenic patients with prominent negative symptoms. Am J Psychiatry, 2001. 158(2): p. 234-43.

17. Paillère-Martinot, M., et al., Cerebral gray and white matter reductions and clinical correlates in patients with early onset schizophrenia. Schizophr Res, 2001. 50(1-2): p. 19-26.

18. Dong, D.B., et al., Reconfiguration of Dynamic Functional Connectivity in Sensory and Perceptual System in Schizophrenia. Cerebral Cortex, 2019. 29(8): p. 3577-3589.

19. Sharma, A., et al., Altered resting state functional connectivity in early course schizophrenia. Psychiatry Res Neuroimaging, 2018. 271: p. 17-23.

20. Penner, J., et al., Higher order thalamic nuclei resting network connectivity in early schizophrenia and major depressive disorder. Psychiatry Res Neuroimaging, 2018. 272: p. 7-16.

21. Penner, J., et al., Temporoparietal Junction Functional Connectivity in Early Schizophrenia and Major Depressive Disorder. Chronic Stress (Thousand Oaks), 2018. 2: p. 2470547018815232.

22. Liu, H., et al., Cigarette smoking and schizophrenia independently and reversibly altered intrinsic brain activity. Brain Imaging Behav, 2018. 12(5): p. 1457-1465.

23. Zhuo, C., et al., Brain structural and functional dissociated patterns in schizophrenia. BMC Psychiatry, 2017. 17(1): p. 45.

24. Peters, H., et al., Changes in extra-striatal functional connectivity in patients with schizophrenia in a psychotic episode. British Journal of Psychiatry, 2017. 210(1): p. 75-82.

25. Wang, H.L., et al., Patients with first-episode, drug-naive schizophrenia and subjects at ultra-high risk of psychosis shared increased cerebellar-default mode network connectivity at rest. Scientific Reports, 2016. 6.

26. Penner, J., et al., Medial Prefrontal and Anterior Insular Connectivity in Early Schizophrenia and Major Depressive Disorder: A Resting Functional MRI Evaluation of Large-Scale Brain Network Models. Front Hum Neurosci, 2016. 10: p. 132.

27. Chen, X., et al., Functional abnormalities of the right posterior insula are related to the altered self-experience in schizophrenia. Psychiatry Research - Neuroimaging, 2016. 256: p. 26-32.

28. Zhou, Y., et al., The selective impairment of resting-state functional connectivity of the lateral subregion of the frontal pole in schizophrenia. PLoS One, 2015. 10(3): p. e0119176.

29. Xu, L., et al., Selective Functional Disconnection of the Dorsal Subregion of the Temporal Pole in Schizophrenia. Sci Rep, 2015. 5: p. 11258.

30. Wang, D., et al., Altered functional connectivity of the cingulate subregions in schizophrenia. Transl Psychiatry, 2015. 5(6): p. e575.

31. Alonso-Solís, A., et al., Resting-state functional connectivity alterations in the default network of schizophrenia patients with persistent auditory verbal hallucinations. Schizophr Res, 2015. 161(2-3): p. 261-8.

32. Zhuo, C., et al., Functional connectivity density alterations in schizophrenia. Front Behav Neurosci, 2014. 8: p. 404.

33. Manoliu, A., et al., Aberrant dependence of default mode/central executive network interactions on anterior insular salience network activity in schizophrenia. Schizophr Bull, 2014. 40(2): p. 428-37.

34. Chang, X., et al., Altered default mode and fronto-parietal network subsystems in patients with schizophrenia and their unaffected siblings. Brain Res, 2014. 1562: p. 87-99.

35. Fan, F.M., et al., Ventral medial prefrontal functional connectivity and emotion regulation in chronic schizophrenia: a pilot study. Neurosci Bull, 2013. 29(1): p. 59-74.

36. Wolf, N.D., et al., Dysconnectivity of multiple resting-state networks in patients with schizophrenia who have persistent auditory verbal hallucinations. Journal of Psychiatry & Neuroscience, 2011. 36(6): p. 366-374.

37. Bluhm, R.L., et al., Spontaneous low-frequency fluctuations in the BOLD signal in schizophrenic patients: anomalies in the default network. Schizophr Bull, 2007. 33(4): p. 1004-12.
